# Multimorbidity and exit from paid employment: the effect of specific combinations of chronic health conditions

**DOI:** 10.1093/eurpub/ckac018

**Published:** 2022-03-07

**Authors:** Gabriel S Gurgel do Amaral, Patricia Ots, Sandra Brouwer, Sander K R van Zon

**Affiliations:** Department of Health Sciences, Community and Occupational Medicine, University of Groningen, University Medical Center Groningen, Groningen, The Netherlands; Department of Health Sciences, Community and Occupational Medicine, University of Groningen, University Medical Center Groningen, Groningen, The Netherlands; Department of Health Sciences, Community and Occupational Medicine, University of Groningen, University Medical Center Groningen, Groningen, The Netherlands; Department of Health Sciences, Community and Occupational Medicine, University of Groningen, University Medical Center Groningen, Groningen, The Netherlands

## Abstract

**Background:**

This study aimed to assess the association between multimorbidity and exit from paid employment, and which combinations of chronic health conditions (CHCs) have the strongest association with exit from paid employment.

**Methods:**

Data from 111 208 workers aged 18–64 years from Lifelines were enriched with monthly employment data from Statistics Netherlands. Exit from paid employment during follow-up was defined as a change from paid employment to unemployment, disability benefits, economic inactivity or early retirement. CHCs included cardiovascular diseases (CVD), chronic obstructive pulmonary disease (COPD), rheumatoid arthritis (RA), type 2 diabetes (T2DM) and depression. Cox-proportional hazards models were used to examine the impact of multimorbidity and combinations of CHCs on exit from paid employment.

**Results:**

Multimorbidity increased the risk of exiting paid employment compared with workers without CHCs (hazard ratio (HR): 1.52; 95% confidence interval (CI): 1.35–1.71) or one CHC (HR: 1.14; 95% CI: 1.01–1.28). The risk for exit from paid employment increased among workers with COPD if they additionally had CVD (HR: 1.39; 95% CI: 1.03–1.88), depression (HR: 1.46; 95% CI: 1.10–1.93) or RA (HR: 1.44; 95% CI: 1.08–1.91), for workers with T2DM if they additionally had CVD (HR: 1.43; 95% CI: 1.07–1.91) or depression (HR: 2.09; 95% CI: 1.51–2.91) and for workers with depression who also had T2DM (HR: 1.68; 95% CI: 1.21–2.32).

**Conclusion:**

This study showed that workers with multimorbidity, especially having a combination of COPD and depression or T2DM and depression, have a higher risk for early exit from paid employment and, therefore, may need tailored support at the workplace.

## Introduction

Over the past decades, the number of workers living with multimorbidity has increased due to ageing as well as better treatment options and prognosis for many chronic health conditions (CHCs).^1^ Multimorbidity is defined as the co-occurrence of two or more CHCs, irrespective of whether one is considered the primary condition.^2^ About 30% of the general European population lives with at least one CHC^3^ and around 23.5% of the European working population has a CHC.^4^^,^^5^ The high prevalence of workers with a single CHC or multimorbidity is a potential problem as CHCs may shorten a person's life span, reduce their quality of life,^5^ and can negatively impact social participation, including reduced participation in the labour market.^6–8^ In Europe, among people aged 50–59 years, 70% of those with one CHC and 52% of those with multimorbidity are employed vs. 74% of those without a CHC.^6^

Early exit from paid employment is a stressful event, especially in case of involuntary exit routes, i.e. unemployment or disability pension.^9^ Previous studies indicate that early exit from paid employment can lead to financial adversities, diminished self-esteem, isolation and disturbed social roles.^10^^,^^11^ In a recent study among older American workers, it was shown that the risk to exit paid employment by receiving disability benefits was almost twice as high for workers with multimorbidity than for workers without a CHC.^7^ The effects of multimorbidity on exit out of paid employment were similar across age groups, gender, marital status and socioeconomic position.^7^ In addition, another prospective study with a 7-year follow-up period conducted in the Netherlands found that the risk of receiving disability benefits was twice as high for workers with multimorbidity when compared with workers with one CHC, and nine times higher when compared with workers without CHCs.^8^ Despite the existing knowledge on the association between multimorbidity and early exit from paid employment, the impact of different combinations of certain CHCs on workers’ exit from paid employment has not been widely investigated yet. Some combinations of CHCs may affect workers’ participation in the labour market more negatively than other combinations or single CHCs. For instance, the combination of physical and mental diseases might be more disabling than having just a physical or mental disorder.^12^ Therefore, a better understanding of how different combinations of CHCs impact early exit from paid employment is needed.

Previous studies have linked some combinations of CHCs to early exit from paid employment and other work-related outcomes, yet literature is scarce, and results are inconsistent. A study conducted among older European workers examined how type 2 diabetes mellitus (T2DM) and cardiovascular diseases (CVD) affected exit from paid employment. No interaction effects were found between T2DM or CVD and comorbidities such as chronic obstructive pulmonary disease (COPD), arthritis or depression.^13^ However, studies focusing on workers with cancer as a primary condition indicated that comorbidities such as CVD or T2DM were more relevant for exiting paid employment than factors related to the cancer itself.^14^^,^^15^ Regarding disability days, a study conducted in the USA examined how the combination of CHCs affected workers. Among adults with two CHCs, those with concomitant T2DM and CVD had the highest increased risk to have disability days, followed by adults with arthritis and CVD.^16^ A better understanding of how specific combinations of CHCs affect exit from paid employment may provide guidance on which workers need tailored prevention and interventions at the workplace.^7^^,^^8^

Therefore, this study aims to examine the association between multimorbidity and exit from paid employment and determine which combinations of CHCs present the highest risk.

## Methods

### Study design

This study uses data from the longitudinal Lifelines Cohort Study enriched with data from Statistics Netherlands. Lifelines is a multi-disciplinary prospective population-based cohort study examining in a unique three-generation design the health and health-related behaviours of 167 729 persons living in the North of the Netherlands. It employs a broad range of investigative procedures in assessing the biomedical, socio-demographic, behavioural, physical and psychological factors which contribute to the health and disease of the general population, with a special focus on multimorbidity and complex genetics.^17^ Participants were recruited between November 2006 and December 2013 through invitations by their general practitioner or family members. In addition, there was an option to self-register. Recruitment and data collection have been described elsewhere.^17^ The study protocol was approved by the medical ethics review committee of the University Medical Center Groningen (2007/152) and conducted in accordance with the Declaration of Helsinki. All participants provided written informed consent upon enrolment.

For the current study, participants were included if they were aged between 18 and 64 years, could be matched with data from Statistics Netherlands and were employed in the month in which they were enrolled in Lifelines. With these restrictions, 27 684 of the 167 729 participants were excluded due to the age restriction, 136 participants could not be matched with Statistics Netherlands, and 28 701 were not employed at baseline. In total, 111 208 participants were included in the current study.

### Measurements

#### Exit from paid employment

Employment status was measured using data from Statistics Netherlands on main income components from Dutch tax registers. Monthly employment data were available from the month of inclusion into Lifelines up to December 2018. Exit from paid employment during follow-up was defined as a change from paid employment to unemployment, disability benefits, economic inactivity or early retirement for at least 3 months.^18^ When a participant reported more than one event of exiting employment over time, only the first event was considered.

#### Chronic health conditions

CHCs were measured at baseline and included CVD, COPD, rheumatoid arthritis (RA), T2DM and depression. These CHCs were included because they have a high burden in terms of disability-adjusted life years (DALYs) and are highly prevalent among workers.^19^^,^^20^ CHCs were classified by a combination of clinical measures, self-reports and medication use based on the Anatomical Therapeutic Chemical (ATC) Classification System. Trained research nurses recorded medication use. Classification of these CHCs was in line with previous studies performed in Lifelines.^21^ From the 5 included CHCs, 10 combination pairs were created. We categorized participants in such a way that groups were not mutually exclusive. In other words, workers with three or more CHCs were included in multiple combination pairs.

#### Covariates

Covariates included age, gender, marital status, educational level, physical activity, smoking, body mass index (BMI), working hours and type of work. Age was used as a categorical variable: 18–34, 35–49 and 50–64 years.^22^ Marital status was categorized into having a partner and not having a partner. Educational level was categorized as low (no education, primary education, lower or preparatory secondary vocational education, junior general secondary education), intermediate (secondary vocational education or work-based learning, senior general secondary education, pre-university secondary education), high (higher vocational education, university education) or other. Physical activity was assessed based on one question from the Short QUestionnaire to ASsess Health enhancing physical activity (SQUASH): ‘On average how many days per week do you cycle, do odd jobs, garden, or exercise for a total of at least half an hour?’.^23^ Participants were classified as sufficiently active if they were active for at least 30 min a day, at least 5 days per week, based on WHO guidelines of 150 minutes of physical activity per week.^24^ Smoking status was categorized as ‘yes’ or ‘no’ with the question ‘do you smoke now, or have you smoked in the past month?’. BMI was calculated using the person’s weight in kilograms divided by the square of the person’s height in metres (kg/m^2^) using anthropometric measurements. Working hours were based on self-report and workers were classified as working full-time (≥36 hours/week) or part-time (<36 hours/week). Type of work was classified as white- or blue-collar work according to the International Standard Classification of Occupations (ISCO)-08.^25^

### Statistical analyses

First, descriptive statistics were used to describe the baseline characteristics of the study population.

Second, the impact of multimorbidity on exit from paid employment was examined with a Cox-proportional hazards model. Hazard ratios (HRs) and corresponding 95% confidence intervals (CIs) for the risk to exit paid employment were presented. The impact of multimorbidity on exit from paid employment was compared with having no CHC and to one CHC. Three models were fitted: Model 1 was unadjusted; Model 2 was adjusted for gender, age, marital status, and educational level; and Model 3 additionally adjusted for physical activity, smoking status, BMI, working hours and type of work. The ‘time at risk’ was defined as the period between enrolment in Lifelines and (i) the last available monthly measure on employment status, (ii) the time until statutory retirement age or (iii) the event ‘exit from paid employment’. We also examined effect modification by age, gender and educational level for the total group of workers by adding interaction terms to the models in which workers with multimorbidity were compared with workers without a CHC and to workers with one CHC.

Third, we examined whether specific combinations of CHCs have a more substantial effect on exit from paid employment when compared with no CHC or one CHC. We compared specific combinations of CHCs with the single CHCs of the combination pair. For example, when a worker had multimorbidity due to COPD and CVD, we compared their risk to exit paid employment to the risk of workers with only COPD and to workers with only CVD. Again, we first performed crude analyses and stepwise adjusted for the covariates. In these models, effect modification was not examined due to insufficient power.

Findings were considered as statistically significant if *P* < 0.05. The proportional hazards assumption was checked by examining Kaplan–Meier plots; no indication for violation of the proportional hazards assumption was found. Analyses were performed using complete cases (missing values ranged from 0.0% for Model 1 to 1.2% for Model 2 and 12.8% for Model 3). IBM SPSS Statistics version 25 was used to perform the analyses.

## Results

### Baseline characteristics

The study population consisted of 111 208 workers. The mean age at baseline was 42.2 (SD 9.6) years, 56.5% was female and 33.7% had a high educational level (table 1). From the total sample, 9.4% had a CHC (*n* = 10 471), and 0.9% had multimorbidity (*n* = 996). In total, 22.4% exited paid employment during follow-up. Workers with multimorbidity were on average older and lower educated than workers without or with one CHC (Supplementary table S1). In addition, 36.9% of workers with multimorbidity exited paid employment, compared with 21.4% of workers without a CHC and 30.8% of workers with one CHC. Workers with depression had the highest risk to exit paid employment, workers with COPD the lowest (Supplementary table S2).

**Table 1:** Baseline characteristics of the total study population (*n* = 111 208)

Characteristic	Mean (SD), median (IQR) or %	*n*
Age (years), mean (SD)	42.2 (9.6)	111 208
Age groups (years) (%)		
18–34	23.2	25 783
35–49	54.6	60 769
50–64	22.2	24 656
Gender (%)		
Female	56.5	62 821
Male	43.5	48 387
Marital status (%)		
Have a partner	88.4	97 236
Not having a partner	11.6	12 799
Educational level (%)		
Low	23.7	26 255
Intermediate	41.1	45 525
High	33.7	37 295
Other	1.5	1673
Specific CHC (%)		
Cardiovascular diseases	1.2	1373
COPD	4.2	4650
Depression	2.5	2815
Rheumatoid arthritis	1.4	1609
Type 2 diabetes	1.9	2121
Number of CHCs (%)		
No CHC	89.7	99 741
One CHC	9.4	10 471
Multimorbidity	0.9	996
Working hours (%)		
Full-time worker (≥36 hours p/w)	47.8	51 520
Part-time worker (<36 hours p/w)	52.2	56 242
Type of work (%)		
White collar	81.5	87 898
Blue collar	18.5	19 948
Exit from paid employment (%)		
Disability benefits	2.3	2603
Unemployment benefits	12.3	13 670
Early retirement benefits	2.4	2658
Economically inactive	5.4	5969
Total number exit from paid employment (%)	22.4	24 900
Time at risk (months), median (IQR)	77 (63–92)	111 208

CHC, chronic health condition; COPD, chronic obstructive pulmonary disease; IQR, interquartile range; SD, standard deviation.

Table 2 shows the combinations of CHCs and the percentage that exits from paid employment. The combinations of COPD with the other CHCs are most prevalent among workers, presenting four combination pairs among the five most frequent. However, the percentage of exit from paid employment is highest for the combinations of CVD or depression with other CHCs.

**Table 2:** Number and percentage of each combination of chronic health conditions for the workers with multimorbidity (*n* = 996)

Combination	*N*	% of people with MM	% exit from work
CVD and COPD	147	14.8	36.1
CVD and depression	66	6.6	45.5
CVD and RA	30	3.0	26.7
CVD and T2DM	160	16.1	39.4
COPD and depression	160	16.1	40.6
COPD and RA	159	16.0	36.5
COPD and T2DM	200	20.1	30.0
Depression and RA	111	11.1	38.7
Depression and T2DM	94	9.4	57.4
RA and T2DM	89	8.9	34.8

Notes: Please note that 95 workers (9.5%) had a chronic health condition (CHC) in addition to the presented combinations (i.e. ≥3 CHCs), as groups were not mutually exclusive.

COPD, chronic obstructive pulmonary disease; CVD, cardiovascular diseases; MM, multimorbidity; RA, rheumatoid arthritis; T2DM, type 2 diabetes mellitus.

### Association between multimorbidity and exit from paid employment

In the fully adjusted model, workers with multimorbidity had an increased risk of exiting paid employment compared with workers without a CHC (HR: 1.52; 95% CI: 1.35–1.71) and compared with workers with one CHC (HR: 1.14; 95% CI: 1.01–1.28) (Supplementary table S3). Effect modification was found for educational level but not for age and gender. Stratified analyses showed that the effect of multimorbidity on exit from paid employment was stronger among workers with a high educational level than among workers with an intermediate or low education (Supplementary table S4).

Table 3 shows the effect of the combinations of CHCs on exit from paid employment. In the fully adjusted model, when compared with workers without a CHC, 6 out of 10 combinations increased the risk of exiting paid employment. The highest risk was observed for the combination of depression and T2DM (HR: 2.78; 95% CI: 2.02–3.83).

**Table 3:** Effect of the 10 possible combination pairs of chronic health conditions on exit from paid employment when compared with having no chronic health condition

	Model 1	Model 2	Model 3
	HR (95% CI)	HR (95% CI)	HR (95% CI)
No CHC	1.00 (ref)	1.00 (ref)	1.00 (ref)
CVD and COPD (*n = *147)	1.95 (1.49–2.56)	1.49 (1.14–1.95)	1.38 (1.03–1.86)
CVD and depression (*n = *66)	3.00 (2.10–4.29)	2.54 (1.76–3.66)	1.79 (1.15–2.77)
CVD and RA (*n = *30)	1.39 (0.69–2.77)	1.04 (0.52–2.07)	1.18 (0.60–2.36)
CVD and T2DM (*n = *160)	2.42 (1.89–3.10)	1.83 (1.42–2.35)	1.73 (1.31–2.29)
COPD and depression (*n = *160)	2.13 (1.67–2.71)	1.84 (1.44–2.36)	1.64 (1.24–2.17)
COPD and RA (*n = *159)	1.91 (1.47–2.47)	1.60 (1.24–2.07)	1.58 (1.19–2.08)
COPD and T2DM (*n = *200)	1.55 (1.20–2.00)	1.28 (0.99–1.65)	1.24 (0.94–1.63)
Depression and RA (*n = *111)	2.10 (1.55–2.83)	1.77 (1.30–2.40)	1.30 (0.88–1.93)
Depression and T2DM (*n = *94)	3.50 (2.68–4.57)	3.09 (2.36–4.04)	2.78 (2.02–3.83)
RA and T2DM (*n = *89)	1.85 (1.30–2.63)	1.47 (1.04–2.10)	1.31 (0.87–1.98)

Notes: Model 1 is not adjusted for covariates; Model 2 is adjusted for age, gender, marital status and educational level; and Model 3 is additionally adjusted for physical activity, smoking, BMI, working hours and type of work. Please note that 95 workers (9.5%) had a chronic health condition (CHC) in addition to the presented combinations (i.e. ≥3 CHCs), as groups were not mutually exclusive.

COPD, chronic obstructive pulmonary disease; CVD, cardiovascular diseases; HRs, hazard ratios; RA, rheumatoid arthritis; T2DM, type 2 diabetes mellitus.

Table 4 shows the risk for exit from paid employment for combinations of CHCs compared with the specific diseases included in the particular pair. In the fully adjusted model, workers with the combination of COPD and CVD (HR: 1.39; 95% CI: 1.03–1.88), COPD and depression (HR: 1.46; 95% CI: 1.10–1.93), and COPD and RA (HR: 1.44; 95% CI: 1.08–1.91) were more likely to exit paid employment than workers with only COPD. Furthermore, workers with the combination of depression and T2DM were more likely to exit paid employment than workers with only depression (HR: 1.68; 95% CI: 1.21–2.32). Finally, workers with the combination of T2DM and CVD (HR: 1.43; 95% CI: 1.07–1.91) and T2DM and depression (HR: 2.09; 95% CI: 1.51–2.91) were more likely to exit paid employment than workers with only T2DM.

**Table 4:** Effect of combination pairs of chronic health conditions on early exit from paid employment when compared with a specific disease from the pair

	Model 1	Model 2	Model 3
	HR (95% CI)	HR (95% CI)	HR (95% CI)
CVD (*n = *1373)	1.00 (ref)	1.00 (ref)	1.00 (ref)
CVD and COPD (*n = *147)	1.08 (0.81–1.44)	0.94 (0.71–1.25)	0.93 (0.68–1.27)
CVD and depression (*n = *66)	1.70 (1.17–2.45)	1.65 (1.14–2.41)	1.14 (0.73–1.79)
CVD and RA (*n = *30)	0.76 (0.38–1.53)	0.65 (0.32–1.31)	0.82 (0.40–1.64)
CVD and T2DM (*n = *160)	1.38 (1.06–1.80)	1.19 (0.91–1.55)	1.24 (0.92–1.67)
COPD (*n = *4650)	1.00 (ref)	1.00 (ref)	1.00 (ref)
COPD and CVD (*n = *147)	1.55 (1.18–2.04)	1.27 (0.96–1.67)	1.39 (1.03–1.88)
COPD and depression (*n = *160)	1.70 (1.32–2.18)	1.59 (1.23–2.04)	1.46 (1.10–1.93)
COPD and RA (*n = *159)	1.52 (1.17–1.97)	1.38 (1.06–1.79)	1.44 (1.08–1.91)
COPD and T2DM (*n = *200)	1.22 (0.94–1.58)	1.08 (0.84–1.40)	1.19 (0.89–1.58)
Depression (*n = *2815)	1.00 (ref)	1.00 (ref)	1.00 (ref)
Depression and CVD (*n = *66)	1.45 (1.01–2.08)	1.35 (0.93–1.96)	1.06 (0.68–1.65)
Depression and COPD (*n = *160)	1.02 (0.79–1.31)	0.98 (0.76–1.26)	0.91 (0.69–1.22)
Depression and RA (*n = *111)	1.00 (0.74–1.36)	0.94 (0.69–1.29)	0.74 (0.50–1.10)
Depression and T2DM (*n = *94)	1.71 (1.30–2.25)	1.68 (1.27–2.21)	1.68 (1.21–2.32)
RA (*n = *1609)	1.00 (ref)	1.00 (ref)	1.00 (ref)
RA and CVD (*n = *30)	0.85 (0.42–1.70)	0.71 (0.35–1.43)	0.96 (0.48–1.93)
RA and COPD (*n = *159)	1.19 (0.91–1.56)	1.13 (0.86–1.48)	1.16 (0.86–1.55)
RA and depression (*n = *111)	1.31 (0.96–1.79)	1.25 (0.91–1.72)	0.93 (0.62–1.39)
RA and T2DM (*n = *89)	1.14 (0.79–1.64)	1.02 (0.71–1.47)	1.02 (0.67–1.56)
T2DM (*n = *2121)	1.00 (ref)	1.00 (ref)	1.00 (ref)
T2DM and CVD (*n = *160)	1.54 (1.19–2.00)	1.32 (1.02–1.72)	1.43 (1.07–1.91)
T2DM and COPD (*n = *200)	0.95 (0.73–1.24)	0.89 (0.69–1.17)	0.92 (0.69–1.23)
T2DM and depression (*n = *94)	2.26 (1.71–2.99)	2.30 (1.74–3.04)	2.09 (1.51–2.91)
T2DM and RA (*n = *89)	1.15 (0.80–1.65)	1.05 (0.73–1.50)	1.00 (0.66–1.53)

Notes: Model 1 is not adjusted for covariates; Model 2 is adjusted for age, gender, marital status and educational level; and Model 3 is additionally adjusted for, physical activity, smoking, BMI, working hours and type of work. Please note that 95 workers (9.5%) had a chronic health condition (CHC) in addition to the presented combinations (i.e. ≥3 CHCs), as groups were not mutually exclusive.

COPD, chronic obstructive pulmonary disease; CVD, cardiovascular diseases; HRs, hazard ratios; RA, rheumatoid arthritis; T2DM, type 2 diabetes mellitus.

## Discussion

This study showed that workers with multimorbidity have an increased risk to exit paid employment compared with workers without a CHC and compared with workers with one CHC. Workers with CVD, depression or RA on top of having COPD had an increased risk of exiting paid employment compared with workers with only COPD. Workers with CVD or depression on top of T2DM had a higher risk of exiting paid employment than workers with only T2DM, and workers with T2DM on top of depression had a higher risk of exiting paid employment than workers with only depression.

Findings from the current study are in line with previous studies showing that multimorbidity increases the risk of exit from paid employment.^7^^,^^8^ A study conducted among US workers showed that workers with multimorbidity have a higher risk to exit paid employment through receiving disability benefits or full retirement than workers without a CHC.^7^ A Dutch study similarly showed that workers with multimorbidity have an increased risk of exiting work through disability benefits when compared with workers with no CHC.^8^ It is important to highlight that both previous studies have investigated the impact of multimorbidity only in adults older than 45 years. The current study included workers from all ages, which enabled us to show that CHCs affect workers from all age groups.

We further found that 6 out of the 10 combinations of CHCs increased the risk of exiting paid employment when compared with workers without a CHC. Two previous Danish studies also investigated the effect of different combinations of CHCs on exit from paid employment.^14^^,^^26^ They showed that comorbidities like CVD and T2DM increased the risk for exit from paid employment among cancer patients more than factors related to cancer itself, such as tumour stage and site.^14^^,^^26^ The impact of multimorbidity, excluding combinations with cancer, on exit from paid employment has not been broadly investigated yet, which limits further comparison of our findings.

The risk for exit from paid employment increased significantly among workers with COPD if they additionally had CVD, depression or RA. CVD and depression similarly increased this risk for workers with T2DM, and T2DM for workers with depression. The high burden of COPD on workers has been demonstrated in previous studies.^19^^,^^20^ Literature links COPD to a lower likelihood of employment when compared with adults without this condition.^27^ In our study, COPD was the most common CHC and presented the lowest risk of exit from paid employment. Additionally, due to the classification of COPD in our study some workers might present a mild COPD, which would not limit them in their work. This high prevalence of COPD combined with the smallest risk of exit could partially explain why three out of four CHCs represented an additional risk when combined with COPD. With regard to the combination of CVD and T2DM, a European study among older workers found that CVD and T2DM increased the risk of exit from paid employment, but they did not find interaction effects between these two CHCs and comorbidities, such as COPD, arthritis or depression.^13^ Previous studies have already shown a strong link between depression and exit from work.^8^^,^^28^ Our finding that the combination of COPD and depression and the combination of T2DM and depression presents a higher risk for exit from paid employment than for COPD or T2DM only may indicate that the combination of physical and mental diseases is more disabling than having just physical or mental disorders.^12^ This is also shown by previous literature for the combination of depression and physical disorders, such as RA or chronic back pain.^12^ Future studies should focus on the burden of having combinations of other mental and physical diseases to be better able to provide targeted help to workers who are most at risk.

Our study has a number of notable strengths. First, by using a combination of objective and self-reported data to classify participants with a CHC, the risk for information bias on the exposure variable is low and more precise compared with studies that based the presence of CHCs completely on self-report.^7^^,^^8^ Second, monthly employment status data were obtained in an objective way through data linkage with Statistics Netherlands, minimizing the possibility of information bias on the outcome variable. Third, because of data linkage with statistics Netherlands on employment status, loss to follow-up was minimal, which limits the possibility of selective drop-out. Fourth, a representative sample from the Northern Netherlands was used which included not only older workers, as in previous studies^7^^,^^8^ but also the working-age population of 18 years of age or older.^29^

This study also has some limitations. First, this study did not account for workers that exited paid employment and returned to work afterwards. Although a large part of older workers may not return to the labour market after exiting paid employment,^30^ this trend might be different for younger age groups. Future studies should try to consider that people potentially re-enter paid employment. Second, our analyses did not take into account that workers could develop CHCs during follow-up since the CHCs were only measured extensively at baseline. By not considering that workers might have developed multimorbidity during follow-up, we may have underestimated the effect of multimorbidity on early exit from paid employment. In addition, we focused on only five CHCs thereby underestimating the total prevalence of CHCs and multimorbidity among the working population. However, the included CHCs are among the non-communicable diseases with the highest amount of DALYs.^19^ Third, despite our large initial sample size, we had too few cases to investigate specific exit routes from paid employment (e.g. into unemployment) in addition to examining specific combinations of CHCs. Fourth, we had no information about psychosocial working conditions, which have been shown to be important for remaining in employment among both workers with and without CHCs.^31^^,^^32^ Finally, we performed multiple tests which may have increased the risk of type 1 error.

The findings from this study have some important scientific implications. First, this study highlights the importance of investigating the presence of multimorbidity when analysing exit from paid employment as multimorbidity has a stronger impact on exit from paid employment than having one CHC. Second, since the reasons for exit from paid employment were not known in the current study, future studies should investigate whether exit from paid employment is indeed related to the CHCs itself or to other reasons.

Findings from this study may also have important implications for policy and practice. Given the increasing number of ageing workers,^33^ combined with the higher probability of developing multimorbidity,^34^ workers, employers, policy makers and healthcare professionals should join forces in the prevention of CHCs and early exit from paid employment. Among workers with CHCs, self-management programs may help them to cope with their disease and workplace health promotion programs have been shown to have positive effects on weight status, eating behaviour and physical activity levels.^35^^,^^36^ In addition, favourable changes in working conditions have been shown to help workers with CHCs to stay in the labour force.^32^ In case of multimorbidity, findings from this study suggest that interventions should be tailored to workers with specific combinations of CHCs as some combinations may pose a greater risk than others for early work exit. Research is needed to examine how specific interventions can contribute to retaining workers with one or more CHCs in the workforce. Future studies could also consider using a weighted multimorbidity index to examine risks of multiple CHCs.

In conclusion, the results from this study show that workers with multimorbidity have a higher risk to exit paid employment than workers without a CHC or than workers with one CHC. The risk for exit from paid employment increased significantly among workers with COPD if they additionally had CVD, depression or RA, and for workers with T2DM if they additionally had CVD or depression, and for workers with depression who also had T2DM. Action is needed to help workers with CHCs maintain their work, especially for workers with COPD that present another CHC and workers with concomitant depression and diabetes.

## Supplementary data

Supplementary data are available at *EURPUB* online.

Key points

- Studies investigating the impact of specific combinations of chronic health conditions (CHCs) on exit from paid employment are lacking.
- The risk to prematurely exit paid employment differs by the specific combination of CHCs.
- Employers and occupational physicians may need to tailor work accommodations according to the specific combinations of CHCs that workers have.

## Supplementary Material

ckac018_Supplementary_Data
