# Supplementary material for: Multimorbidity and exit from paid employment: the effect of specific combinations of chronic health conditions
Source: Eur J Public Health. 2022 Mar 7;32(3):392–7. doi: 10.1093/eurpub/ckac018 (PMC9159305; doi:10.1093/eurpub/ckac018)
Supplement: ckac018_Supplementary_Data [file ckac018_supplementary_data.docx]

**Supplementary table 1.** Baseline characteristics of the study population (n= 111,208) per number of CHCs (no, one CHC or MM)

|  | No CHC |  | One CHC | | MM |  |
| --- | --- | --- | --- | --- | --- | --- |
| Characteristic | Mean (SD),  median (IQR) or % | n | Mean (SD),  median (IQR) or % | n | Mean (SD),  median (IQR) or % | n |
| Age (years), mean (SD) | 41.6 (9.5) | | 46.8 (8.5) | | 50.0 (7.3) | |
| Age groups (years), (%)  18-34 35-49 50-64 | 24.9  54.6  20.5 | 24,851  54,492  20,398 | 8.7  55.4  35.9 | 921  5,800  3,759 | 2.0  47.9  50.1 | 20  477  499 |
| Gender, (%)  Female  Male | 56.6  43.4 | 56,487  43,254 | 55.6  44.4 | 5,825  4,646 | 51.1  48.9 | 509  487 |
| Marital status, (%)  Have a partner  Not having a partner | 88.6  11.4 | 11,268  87,436 | 86.3  13.7 | 8,935  1,415 | 88.2  11.8 | 865  116 |
| Educational level, (%) Low Intermediate  High  Other | 22.2  41.4  35.0  1.5 | 22,002  41,075  34,776  1,473 | 36.0  39.4  22.9  1.7 | 3,759  4,105  2,386  179 | 49.7  34.7  13.4  2.1 | 494  354  133  21 |
| Specific CHC, (%)  Cardiovascular diseases  COPD  Depression Rheumatoid arthritis  Type 2 diabetes |  |  | 9.9  38.9  23.3  12.1  15.8 | 1034  4,068  2,444  1,271  1,654 | 34.0  58.4  37.2  33.9  46.9 | 339  582  371  338  467 |
| Exit from paid employment, (%)  Disability benefits  Unemployment benefits  Early retirement benefits  Economically inactive | 2.0  11.9  2.1  5.3 | 1,993  11,888  2,132  5,295 | 4.8  15.6  4.6  5.8 | 499  1,638  477  610 | 11.1  14.5  4.9  6.4 | 111  144  49  64 |
| Total exit from paid employment, (%) | 21.4 | 21,308 | 30.8 | 3,224 | 36.9 | 368 |
| Time at risk (months), median (IQR) | 77 (64, 92) | | 75 (47, 92) | | 69 (30, 87) | |

Notes: SD – standard deviation; CHC – chronic health condition; COPD – chronic obstructive pulmonary disease; IQR – interquartile range; MM - multimorbidity.

**Supplementary table 2.** The impact of specific CHCs on early exit from paid employment compared to having no CHC.

|  | **Model 1** | **Model 2** | **Model 3** |
| --- | --- | --- | --- |
|  | HR (95% CI) | HR (95% CI) | HR (95% CI) |
| No disease (n=99,741) | 1.00 (ref) | 1.00 (ref) | 1.00 (ref) |
| CVD (n=1,373) | 1.82 (1.66; 1.99) | 1.57 (1.43; 1.72) | 1.53 (1.38; 1.70) |
| COPD (n=4,650) | 1.28 (1.21; 1.35) | 1.17 (1.11; 1.24) | 1.14 (1.07; 1.22) |
| Depression (n=2,815) | 2.09 (1.97; 2.22) | 1.86 (1.75; 1.98) | 1.71 (1.59; 1.83) |
| RA (n=1,609) | 1.63 (1.50; 1.78) | 1.43 (1.31; 1.57) | 1.36 (1.23; 1.50) |
| T2DM (n=2,121) | 1.62 (1.50; 1.75) | 1.41 (1.30; 1.52) | 1.37 (1.26; 1.50) |

Notes: CVD – cardiovascular disease; COPD – chronic obstructive pulmonary disease; RA – rheumatoid arthritis; T2DM – type 2 diabetes mellitus; HR, hazard ratio; CHC, chronic health condition; Model 1 is not adjusted for covariates, model 2 is adjusted for age, gender, marital status and educational level, and model 3 is additionally adjusted for physical activity, smoking, BMI, working hours and type of work.

**Supplementary table 3.** Effect of multimorbidity on exit from paid employment when compared to no CHC or one CHC.

|  | **Model 1** | **Model 2** | **Model 3** |
| --- | --- | --- | --- |
|  | HR (95% CI) | HR (95% CI) | HR (95% CI) |
| No CHC (n=99,741) | 1.00 (ref) | 1.00 (ref) | 1.00 (ref) |
| Multimorbidity (n=996) | 2.01 (1.81; 2.23) | 1.66 (1.50; 1.85) | 1.52 (1.35; 1.71) |
|  |  |  |  |
| One CHC (n=10,471) | 1.00 (ref) | 1.00 (ref) | 1.00 (ref) |
| Multimorbidity (n=996) | 1.32 (1.18; 1.46) | 1.20 (1.08; 1.34) | 1.14 (1.01; 1.28) |

Notes: HR, hazard ratio; CHC, chronic health condition; MM, multimorbidity; Model 1 is not adjusted for covariates, model 2 is adjusted for age, gender, marital status and educational level, and model 3 is additionally adjusted for physical activity, smoking, BMI, working hours and type of work.

**Supplementary table 4.** Effect of multimorbidity on exit from paid employment, stratified by educational level, when compared to no CHC or one CHC.

|  | High education | Intermediate education | Low education |
| --- | --- | --- | --- |
|  | HR (95% CI) | HR (95% CI) | HR (95% CI) |
| No CHC (n=99,741) | 1.00 (ref) | 1.00 (ref) | 1.00 (ref) |
| Multimorbidity (n=996) | 2.05 (1.52; 2.78) | 1.47 (1.19; 1.81) | 1.45 (1.23; 1.72) |
|  |  |  |  |
| One CHC (n=10,471) | 1.00 (ref) | 1.00 (ref) | 1.00 (ref) |
| Multimorbidity (n=996) | 1.52 (1.12; 2.08) | 1.02 (0.82; 1.26) | 1.19 (1.00; 1.41) |

Notes: HR, hazard ratio; CHC, chronic health condition; MM, multimorbidity. Model is adjusted for age, gender, marital status and educational level, physical activity, smoking, BMI, working hours and type of work.
